# Community health worker in hard-to-reach rural areas of Myanmar: filling primary health care service gaps

**DOI:** 10.1186/s12960-016-0161-4

**Published:** 2016-10-21

**Authors:** Angkana Sommanustweechai, Weerasak Putthasri, Mya Lay Nwe, Saw Thetlya Aung, Mya Min Theint, Viroj Tangcharoensathien, San Shway Wynn

**Affiliations:** 1 International Health Policy Program (IHPP), Ministry of Public Health, Nonthaburi, Thailand; 2 Ministry of Health, the Republic of the Union of Myanmar, Naypyidaw, Myanmar; 3 WHO Country Office Myanmar, Yangon, Myanmar

**Keywords:** Community health worker, Primary health care, Myanmar, Hard-to-reach townships

## Abstract

**Background:**

Myanmar is classified as critical shortage of health workforce. In responses to limited number of trained health workforce in the hard-to-reach and remote areas, the MOH trained the Community Health Worker (CHW) as health volunteers serving these communities on a pro bono basis. This study aimed to assess the socio-economic profiles, contributions of CHW to primary health care services and their needs for supports to maintain their quality contributions in rural hard to reach areas in Myanmar.

**Methods:**

In 2013, cross-sectional census survey was conducted on all three groups of CHW classified by their training dates: (1) prior to 2000, (2) between 2000 and 2011, and (3) more recently trained in 2012, who are still working in 21 townships of 17 states and regions in Myanmar, using a self-administered questionnaire survey in the Burmese language.

**Findings:**

The total 715 CHWs from 21 townships had completely responded to the questionnaire. CHWs were trained to support the work of midwives in the sub-centres and health assistant and midwives in rural health centres (RHCs) such as community mobilization for immunization, advocates of safe water and sanitation, and general health education and health awareness for the citizens. CHWs were able to provide some of the services by themselves, such as treatment of simple illnesses, and they provided services to 62 patients in the last 6 months. Their contributions to primary health care services were well accepted by the communities as they are geographically and culturally accessible. However, supports from the RHC were inadequate in particular technical supervision, as well as replenishment of CHW kits and financial support for their work and transportation. In practice, 6 % of service provided by CHWs was funded by the community and 22 % by the patients. The CHW’s confidence in providing health services was positively associated with their age, education, and more recent training. A majority of them intended to serve as a CHW for more than the next 5 years which was determined by their ages, confidence, and training batch.

**Conclusions:**

CHWs are the health volunteers in the community supporting the midwives in hard-to-reach areas; given their contributions and easy access, policies to strengthen support to sustain their contributions and ensure the quality of services are recommended.

**Electronic supplementary material:**

The online version of this article (doi:10.1186/s12960-016-0161-4) contains supplementary material, which is available to authorized users.

## Background

Given the recent positive economic performance, the Republic of the Union of Myanmar is classified as a lower middle-income country with a gross national income per capita of US$ 1280 in 2014 [1].

Government health expenditure had increased from the very low level of 1 % of total government expenditure in 2005 to 1.5 % in 2012 [2], despite the strong political commitment on health, but this level is still low in response to the health needs of the people. The embargo in the past decades resulted in limited flow of official development assistance (ODA) to Myanmar, until the late 1990s, and then, ODA reached its peak in 2009 in response to Cyclone Nargis [3].

Myanmar is 1 of the 57 countries classified as having a critical shortage of health workforce, only 1.61 doctors, nurses, and midwives per 1000 population [4]. It was far below the global benchmark of 2.28 doctors, nurses, and midwives per 1000 population, which is a level that would provide adequate coverage of essential health services to the people [5].

The health workforce maldistribution exacerbates the shortage problems; it jeopardizes access to services resulting in poorer health status among people living in hard-to-reach areas. Limited access to functioning health services jeopardizes the achievement of health-related Millennium Development Goals (MDGs). By 2015, Myanmar was not on track for all health-related MDGs, especially on the reduction in infant and child mortality. Large efforts are required to reduce this rate; now the under-five mortality rate and infant rate stood at 51 and 40 per 1000 live births, respectively, in 2015 [6].

Task shifting is defined as the delegation of some tasks to less specialized volunteers [7]. This was commonly practised in countries facing a critical shortage of the health workforce. Dating back to the 1978 Alma Ata Declaration, countries had proven the significant contribution of village health volunteers [8].

**Fig. 1:**
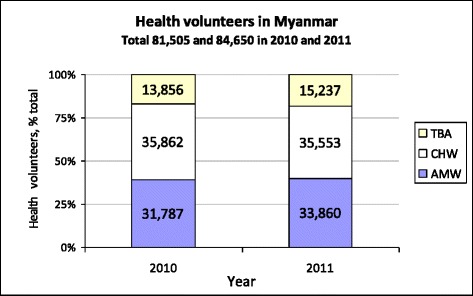
Health volunteers in Myanmar. Source: Public Health Statistics, 2010–2011

The Ministry of Health (MOH) classifies health volunteers serving rural communities in their voluntary capacity into three cadres: traditional birth attendants (TBAs), auxiliary midwives (AMWs), and community health workers (CHWs) [9]. All were trained to support the work of basic health staff (BHS) posted in rural communities. BHS are government officials, including health assistants, lady health visitors, midwives, and public health supervisors grades I and II. In 2010 and 2011, there were a total of 81 505 and 84 650 volunteers in Myanmar, respectively. Of these, CHWs were the majority, consisting of 44 and 42 % of the total, followed by AMWs, consisting of 39 and 40 % of the total volunteers, respectively (see Fig. 1). In addition to BHS, these volunteers are auxiliary health workforce assets in the communities requiring policy attention to maximize their contributions.

In response to the limited number of trained health workforce in the hard-to-reach and remote areas, the CHW is one of the three cadres of health volunteers serving these communities, supporting the work of BHS, especially midwives on a pro bono basis. The MOH trained them using different sources of funding, either from itself or international development partners when opportunities arose. Where budget is available, some got refresher courses.

CHWs are nominated by a village health committee. By the existing system, both females and males can be recruited as CHWs in Myanmar, without intentional gender discrimination in the recruitment process. All AMWs were female [10]; both of these were recruited from the same community. The criteria include a person who is interested in delivering health care and messages to the rural community, preferably those who are under the age of 35, having middle school-level education at least, and living in the rural area, but not the village where a sub-centre exists, in order to have sufficient education to read and write the Burmese language and speak the local dialect. The final selection of CHWs is done at the township level. After that, they are trained by the training team members from townships who have already attended the training for trainers, jointly with TMO, and sometimes, trainers from their respective state/region joined the training as trainers or facilitators.

The Global Alliance on Vaccine and Immunization (GAVI) not only supported Myanmar in introducing new and under-used vaccines, it also supports health system strengthening (HSS) by recruitment, training new batches and providing refresher courses for the current CHWs in the GAVI HSS programme-supported townships. Due to resource constraints, each township has a quota of 20 new recruits for a 1-month CHW training and 50 refresher courses for the older CHW batches. As a part of routine monitoring and evaluation of the GAVI HSS project in Myanmar, the assessment of the CHW’s contribution to fill the gap of primary health services in hard-to-reach areas is conducted. This study aimed to assess their profiles, their contributions and the needed support in order to improve the quality of their work, and their intention to serve the communities in the next 5 years.

## Methods

A quantitative self-administered questionnaire survey was applied. This study is a part of the routine monitoring and evaluation of the GAVI HSS project in Myanmar. The Ministry of Heath, the Republic of the Union of Myanmar, had waived the ethical clearance (Additional file 1). The questionnaire was developed in the English language by a research team based on the questions related to the CHW’s contributions and their need for supports to maintain their quality contributions. Then it was translated into Burmese.

The contents of the questionnaire were divided into three parts. The first part was on the CHW’s personal background, including age, marital status, education, domicile if they live in the village they served, and their main job. The second part covered their history of being a CHW: who proposed them for training, their main motivation, and when were they first trained (1-month course) or their most recent 5-day refresher course. The respondents were selected based on training dates: the recent batch of GAVI trained in 2012, the older batches who were trained between 2000 and 2011, and the oldest batch who were trained prior to 2000, and their confidence to provide service to villagers. The third part was on their contributions to villagers, which included environment health such as advice on water and sanitation, general health education to communities, community mobilization for immunization, treatment of simple illnesses, and referral of serious illness cases and how many refused referral (mostly based on financial reasons that the households could not afford to pay). Their need for supports such as incentives for their work, transport, and/or refresher training; their perception of being accepted by the communities; financial support they got from communities and patients they served; and satisfaction with the supports from the rural health centre (RHC) and sub-centre. This part ended with the number of years they intended to serve the communities and reasons for quitting as a CHW.

The first draft questionnaire was tested on the relevance of contents to the CHW in the catchment areas of two selected rural health centres in two townships, one as the best and the other the worst programme performance in the view of the township medical officer. A total of 30 CHWs responded to the pilot study in the Burmese version.

After the pilot, the contents in the questionnaire were reviewed, revised, and finalized. The final English version was translated into a Burmese version and distributed to 19 GAVI HSS townships and two new townships planned for 2014 implementation (Pinlong and Thanatpin). As the systematic random sampling was not possible to manage, this study decided to apply a census survey where all CHWs irrespective of their training period and funding sources who were working in these 21 townships were all recruited as samples.

The questionnaires were sent out to the BHS of all health facilities in these 21 townships at the July 2013 township monthly meeting. The BHS circulated the questionnaires to all CHWs in their catchment area after the meeting. After reading and signing the consent form on the first page of the questionnaire, respondents were requested to answer the questionnaires independently. The BHS collected the completed questionnaires.

The completed questionnaires were collected from the BHS at the August 2013 township monthly meeting. All questionnaires were sent to the World Health Organization (WHO) country office in Yangon by pouch. Data was entered into Microsoft Excel, and analysis was conducted using STATA. The descriptive statistics were applied including multiple logit regression.

## Results

### Profiles of CHW

A total of 715 CHWs from 21 townships completely responded to the questionnaire, and response rate was 100 %. All were men, 30 % of them age more than or equal to 50 years, and 34 % were single. On their educational background, a majority of them, nearly 80 %, were grades 9 and 11, while 6 % was bachelor or above. Almost all, 98 %, of CHWs lived in the village they provided services to and spoke the same dialect as the villagers. The CHW is a voluntary job, and 73 % of them had to earn a living from farming, 14 % are shopkeepers, and 13 % had other occupations.

### About being a CHW

More than half of the CHWs, 53 %, reported that they applied for CHW training on their own, while 21 % were proposed by the midwives in the sub-centre, 15 % were nominated by the village head, and 11 % by the villagers. Altruism was the main motivation to be a CHW, as 87 % reported the chance to serve the people in their own villages, 9 % said they were recognized by the communities, and less than 1 % reported having a chance to earn some money from being a CHW.

Of the total sample of CHWs, 32 % were recently trained in a 1-month course in the GAVI HSS programme in 2012, while 40 % were the oldest batch that was trained prior to 2000, and 28 % were trained between 2000 and 2011 by multiple sources of funding. In the GAVI HSS programme, there was a 5-day refresher course offered to the existing CHWs, and 55 and 47 % of those trained between 2000 and 2011 and before 2000, respectively, reported having a refresher course.

About their confidence in providing services, 31 % reported very confident, while 58 % reported confident, and 10 % reported fairly confident. Less than half percent reported they are “not confident”.

### The contributions of CHW and needed supports

In the last 6 months prior to the survey, 95 % reported they advocated the environment health such as clean water and safe sanitation, 94 % reported support provision of health education to villagers, 96 % reported support community mobilization for immunization of children under the age of 1 year, and 83 % reported having offered treatment of minor illnesses, with an average of 62 cases treated during the last 6 months. They reported an average 12 serious illnesses identified and referred during the last 6 months, but around half of them, 6 cases, refused the referral despite eligibility to the Hospital Equity Fund for fear of unexpected expenditure, which they could not afford. Clearly, CHWs had supported and contributed to community health services.

A majority, 57 %, reported satisfied and 29 % reported they were very satisfied with the supports they received from the sub-centre or RHC. Almost all of the CHWs reported the importance of technical supervision by midwives and other BHS as well as refresher course; 80 % needed financial support for transport to reach villagers while 71 % needed replenishment of CHW kits including consumable items such as sanitary soap and a range of basic drugs like paracetamol, mebendazole, and phenoxymethylpenicillin; and they also needed financial support for their work.

Of the total CHWs, 69 % reported they were well accepted by the community while only a tiny fraction as not being accepted. A few key problems encountered by CHWs are a lack of health awareness among the people (83 %) and low education and lack of health literacy among women (70 %). There were 6 % of respondents who reported they got financial supports from the community while 22 % got financial rewards from the patients they served (Table 1).

**Table 1:** Contribution of CHW and their needed support

Contributions of CHW	Frequency	Percent
• Environmental health	595	95
• Health education	571	94
• Community mobilization for EPI	479	83
• Treatment of minor illness	9.2	34
Contributions of CHW	Mean	Standard error
• Number of cases with minor illness	61.8	139
• Number of cases with serious illness who were referred	11.8	25
• Number of cases with serious illness who refused referral	6.1	10
Support to CHW by BHS and sub-centres	Frequency	Percent
• Very satisfied	202	29
• Satisfied	401	57
• Fairly satisfied	63	9
• Unsatisfied	27	4
• Very unsatisfied	5	1
Needed support		
• Technical supervision	638	99
• Refresher course	620	99
• Kit replenishment	537	94
• Financial support for transportation	430	80
• Financial support for work	369	71
Travelling difficulty to hard-to-reach areas		
• Having difficulty	424	68
• Not having difficulty	201	32
Experiencing patients refusing to be referred to higher facilities		
• Ever experienced	309	58
• Never experienced	225	42
Sources of financial assistance		
• From community fund		
○ Ever received	40	6
○ Never received	601	94
• From individual patients		
○ Ever received	133	22
○ Never received	481	78

### CHW: confidence and intention to stay more than 5 years

A futuristic question was asked on how many years they intended to serve as a CHW. A majority, 87 %, reported an intention to serve more than 5 years, 13 % intended to serve between 3 and 5 years, 8 % between 1 and 3 years, and 2 % will serve less than a year. Potential reasons for quitting were as follows: 28 % said when moving out of the village, 26 % said if they feel they could not contribute as much as they expected, 15 % when getting a permanent job or other full-time employment, a similar percentage if they felt not proud of their work, and only 7 % said when getting married.

Table 2 showed multiple logit regression; the confidence of offering services to the community was a dependent variable, whereas independent variables included age group, education level (grades 5, 9, 11, and bachelor or above), recruitment pattern (self-application, proposed by midwives, villagers, and village head), and batch of training (before 2000, 2000–2011, and 2012).

**Table 2:** Factor contributing to the confidence of providing services by CHWs (multiple logit regression)

Feeling confident	Odds ratio	Std. err.	*P* value	95 % CI	
Age (compared with youngest, <20)					
• 20–29	1.545	0.661	0.308	0.669	3.572
• 30–39	2.511	1.216	0.057	0.972	6.487
• 40–49	6.363	4.026	0.003	1.841	21.992
• ≥50	7.442	4.608	0.001	2.212	25.045
Education (compared with grade 5)					
• Grade 9	1.013	0.475	0.978	0.404	2.539
• Grade 11	1.125	0.537	0.805	0.442	2.867
• Bachelor or above	0.950	0.608	0.936	0.271	3.331
Recruitment (VS self-application)					
• Local midwives	0.944	0.307	0.859	0.499	1.786
• Villagers	1.450	0.734	0.463	0.538	3.912
• Village head	0.798	0.303	0.552	0.379	1.679
Training batch (compared with batched trained before 2000)					
• 2000–2011	1.037	0.442	0.933	0.449	2.393
• 2012	1.269	0.588	0.606	0.512	3.145

Note: pseudo *R* ^2^ = 0.0604

The regression analysis showed that the older the CHW, the higher their confidence to provide services (with an odds ratio of 7.44 for those aged more than 50 years old with statistical significance). CHWs who were grade 11, those who were nominated by villagers, and also the recent batch trained in 2012 reported higher confidence but not statistically significant.

Table 3 showed multiple logit regression; the intention to continue their contribution for more than 5 years was a dependent variable, whereas independent variables were similar to the above as well as the level of confidence in providing services. The regression analysis showed that CHWs with ages 40–49 had the highest probability of intention to contribute more than 5 years (OR = 7.4) with statistical significance. Also, the high level of confidence had a higher probability of serving the community more than 5 years (OR = 1.8). CHWs who were grade 9, those who were nominated by villagers, and an older batch trained before 2000 reported higher probability of serving more than 5 years with statistical significance.

**Table 3:** Factor contributing to the intention to contribute more than 5 years service by CHW (multiple logit regression)

Intention to serve communities >5 years	Odds ratio	Std. err.	*P* value	95 % CI	
Confidence in providing services (VS not confident)	1.838	0.648	0.084	0.921	3.669
Age (compared with youngest, <20)					
• 20–29	2.353	0.912	0.027	1.101	5.029
• 30–39	4.914	2.397	0.001	1.889	12.783
• 40–49	7.374	4.795	0.002	2.062	26.373
• ≥50	2.683	1.476	0.073	0.912	7.889
Education (compared with grade 5)					
• Grade 9	1.037	0.537	0.943	0.376	2.862
• Grade 11	0.656	0.330	0.403	0.245	1.761
• Bachelor or above	0.745	0.484	0.650	0.209	2.659
Recruitment (VS self-application)					
• Local midwives	0.383	0.115	0.001	0.212	0.690
• Villagers	2.057	1.311	0.258	0.590	7.173
• Village head	0.568	0.230	0.163	0.257	1.256
Training batch (compared with batched trained before 2000)					
• 2000–2011	0.466	0.224	0.112	0.182	1.194
• 2012	0.308	0.153	0.018	0.116	0.815

Note: pseudo *R* ^2^ = 0.1623

## Discussion

This study was the first assessment of the contributions by CHWs in hard-to-reach areas in Myanmar. MOH trained CHWs to support basic health services while female AMWs aimed to support maternal and child health services. Male CHWs’ fit for purposes such as disease control, sanitation, social mobilization supporting immunization, and health education. About one third (31 %) are 50 years old or more. Age also related to the confidence to provide services: the older the CHW, the higher the confidence they have in service provision.

The key motivations were altruism in servicing people in their home village. Despite being volunteers, CHWs received financial support from patients and the community in particular for transport cost for outreach to households as there is no funding support by the MOH. Coherently, the communities recognized their values, as almost all reported they were well accepted by villagers. CHWs play a bridging role between local communities and the health facilities through supporting health promotion and referral of severe cases to township hospitals in order to save lives. Their strengths are physical accessibility, cultural sensitivity, and language-friendly services for the local people [11]; they lived in the village they served and spoke the same local dialect.

CHWs reported a few challenges such as lack of health awareness and literacy especially among female villagers. Maternal literacy is a more important factor than education especially in low-income families, contributing to child survival [12–14].

Half of the patients diagnosed with serious illness denied referral even though the transport cost and treatment fees at township hospitals would be fully covered by the Hospital Equity Fund. Typically, the transportation is a common barrier to access health services, in particular among the rural poor [15, 16]. Even though transport and medical fees are covered, there are other associated financial barriers such as food and lodging for accompanying relatives.

Shifting some of these tasks from BHS to less skilful CHWs allows improved health coverage in hard-to-reach communities. Task shifting is commonly applied by various countries in response to the health needs of the people in the context of resource-poor settings; in some cases, these volunteers improve quality and efficiency [17]. Ethiopia deployed 30 000 new health extension workers to provide a limited package of priority preventive interventions to the vast majority of rural people. The low skill among these volunteers is recognized as a limiting factor [18]. Though Ethiopian extension workers had up-scaled to beyond grade 10 candidates, 80 % of Myanmar CHWs were grades 9 and 11.

Factors influencing CHW productivity and retention are important for programme design and strengthening. Their close relationship with health workers and the community are important factors; others are recognition by and support from the community, adequate supervision and incentives from the government, and the community values the CHW contributes [19, 20]. In this study, CHWs reflected a strong need for technical supervision and support from midwives, replenishment of CHW kits, refresher courses, and financial support for their transport cost outreach to the households. With the current fiscal space in Myanmar, all CHWs and other health volunteers are not covered by the MOH payroll [21].

A number of limitations are identified. First, a self-administered questionnaire does not allow objective assessment of their own performances. Second, this study does not cover perspectives from their supervisors, BHS, and midwives, on their quality and competencies. Third, the study confined CHWs working in hard-to-reach communities in GAVI-supported townships; hence, it cannot be generalized with other normal-setting townships. Despite these limitations, this study demonstrates a critical and potential role of CHWs in improving health in rural Myanmar.

## Conclusion

CHWs provide significant support to the BHS and are considered the most valuable rural assets to deliver primary health care services to people normally not accessible to township hospital and doctor services and adequate health prevention and promotion. The MOH needs to include CHWs and other health volunteers into the national HRH strategic plan, with an appropriate plan for training, refreshing, support, and supervision. In the context of commitment to the Sustainable Development Goals (SDGs), these volunteers are essential in supporting achievement of the SDGs.

Decision to compensate their travel allowances depends on the MOH fiscal space, though naturally some of these costs are covered by the communities and patients. Social recognition such as through an annual medal for the best dedicated CHW and other health volunteers are important non-financial incentives and can be convened by the MOH. Retraining, supervision, and support will prevent them from becoming a “quack” while maximize their potential contributions.

## Additional file

Additional file 1: Letter of waiver. (PDF 681 kb)

## Abbreviations

AMWs: Auxiliary midwives
BHS: Basic health staff
CHW: Community health worker
GAVI: Global Alliance on Vaccine and Immunization
HSS: Health system strengthening
MDGs: Millennium Development Goals
MOH: Ministry of Health
ODA: Official development assistance
RHCs: Rural health centres
TBAs: Traditional birth attendants
WHO: World Health Organization
