# Supplementary material for: Community health worker in hard-to-reach rural areas of Myanmar: filling primary health care service gaps
Source: Hum Resour Health. 2016 Oct 21;14:64. doi: 10.1186/s12960-016-0161-4 (PMC5075211; doi:10.1186/s12960-016-0161-4)
Supplement: Additional file 1: — Letter of waiver. (PDF 681 kb) [file 12960_2016_161_MOESM1_ESM.pdf]

To Whom it May concern

Dear Sir/Madam,

The assessment of GAVI Health System Strengthening Program in the implementing townships was jointly conducted by Ministry of Health Myanmar, Staffs from GAVI HSS section of WHO country office Myanmar and International Health Policy Program of the Ministry of Public Health of Thailand. As this is part of regular monitoring and evaluation of key programs in Myanmar, and it is not a research as such, hence an ethical clearance was waived for the surveys and the assessment of the GAVI HSS program.

with regards,

23/2/16

Dr Wai Mar Mar Htun

ဒေါက်တာဝေမာမာထွန်း
လက်ထောက်အတွင်းဝန်
ကျန်းမာရေးဝန်ကြီးဌာန

Assistant Secretary

Ministry of Health

Myanmar

Date: 23rd February 2016
